# An experimental study on the effect of symptom expectations on mental fatigue and motivation in people with primary biliary cholangitis

**DOI:** 10.1038/s41598-025-16191-2

**Published:** 2025-08-26

**Authors:** Laura Buck, Jule Herzberg, Bert Lenaert, Bernd Löwe, Johannes Hartl, Christoph Schramm, Anne Toussaint

**Affiliations:** 1 Department of Psychosomatic Medicine and Psychotherapy, University Medical Center Hamburg-Eppendorf, Hamburg, Germany; 2 Department of Life Span Psychology, Faculty of Psychology, Open University, Heerlen, The Netherlands; 3 Department of Health Promotion, Faculty of Health, Medicine and Life Sciences, Maastricht University, Maastricht, The Netherlands; 4 I. Department of Medicine, University Medical Center Hamburg- Eppendorf, Hamburg, Germany; 5 Martin Zeitz Centre for Rare Diseases, University Medical Center Hamburg-Eppendorf, Hamburg, Germany; 6 Hamburg Center for Translational Immunology (HCTI), Hamburg, Germany

**Keywords:** Fatigue, Motivation, Symptom expectations, Working memory, Autoimmune liver disease, Psychology, Hepatology

## Abstract

Fatigue is the most common symptom in people with primary biliary cholangitis (PBC) and resistant to current treatment modalities. The aim of this study was to investigate the effect of negative and positive fatigue expectations in people with PBC on experienced fatigue and the motivational urge to stop a cognitive task. A subsample of the SOMA.LIV study of *N* = 46 people with PBC was randomly assigned to two experimental conditions. They received either fatigue-inducing or fatigue-reducing task instructions for a subsequent cognitive task. Participants rated their fatigue expectations prior to the task and their fatigue and motivational urge to stop after each of five task blocks. The total sample showed an increase in subjective fatigue and urge to stop across all task blocks. Participants receiving fatigue-inducing task instructions reported higher urge to stop compared to the group with fatigue-reducing task instructions. Both groups did not differ significantly in fatigue expectations and subjective fatigue. Our findings suggest that people with PBC may benefit from encouragement to engage in cognitive activities and maintain mental effort by verbal suggestions - an effect that can be of use in clinical practice to reduce potential avoidance behaviour.

## Introduction

Primary biliary cholangitis (PBC) is a chronic inflammatory liver disease characterised by immune-mediated destruction of the intrahepatic bile ducts^1^. In the absence of effective treatment, the progressive destruction of bile ducts can lead to cholestasis, fibrosis progression and eventually cirrhosis which may require liver transplantation^2^. A systematic review of population-based studies has revealed an incidence rate of 0.5 to 5.8 persons/ year with a prevalence of 1.91 to 40.2 per 100.000 persons across Europe, North America, Asia, and Australia^3^. 90% of patients are female with the majority being diagnosed between the 4th and 6th decade of life^4^. The diagnosis of PBC is often preceded by an asymptomatic elevation of liver enzymes and eventually confirmed by chronically elevated cholestasis parameters (i.e. alkaline phosphatase) in combination with elevated levels of antimitochondrial antibodies (AMA) and, less often, histological examination^5^. The aetiology of PBC is incompletely understood with a combination of genetic predisposition and external environmental triggers (e.g. history of urinary tract infections, past smoking, exposure to toxic substances) being discussed as etiological mechanisms^5–7^. In the course of PBC, up to 70% of people develop accompanying physical symptoms such as fatigue, sicca or pruritus^8^. These symptoms often interfere with daily activities, severely impair their quality of life and may result in higher self-reported depressive and anxiety symptoms^9–12^. Fatigue is the most common symptom in PBC with more than 40% of patients reporting moderate to severe levels^12^. It is experienced by the people affected as the symptom with the greatest impact on their quality of life^13^. The intake of ursodeoxycholic acid (UDCA) as the first line treatment in PBC has shown its effectiveness in preventing disease progression and improving transplant-free survival^14^, whereas it does not alleviate people from their fatigue symptoms^15^. These have been found to be largely unrelated to the stage of liver disease^16^, may persist even after liver transplantation^17^ and are often associated with depressive symptoms, autonomic dysfunction, and sleep disturbances^18^. To date, the mechanisms underlying fatigue evolvement and maintenance in people with PBC are largely unknown^18,19^. Elevation of inflammatory cytokines or progesterone metabolites have been postulated as underlying mechanisms of fatigue in PBC and other chronic inflammatory conditions such as rheumatoid arthritis^20,21^. At the behavioural level, a meta-analysis among individuals with chronic diseases has shown a small positive association between fatigue severity and avoidance behaviour (general and fatigue-specific avoidance) implying that this might be a target for behavioural interventions^22^. Avoidance behaviour is a protective strategy in situations that had previously elicited somatic symptoms and is often triggered by precise expectancies about somatic symptoms such as pain, itch or fatigue^23^. Negative symptom expectations may manifest as catastrophising thoughts and negative affect, further intensifying avoidance behaviour and increasing psychological distress^23^. The influence of symptom expectations has so far been studied in the context of placebo and nocebo effects with a specific focus on pain perception and has recently been extended to other symptoms such as itch and fatigue^23–25^. In etiological models of persistent somatic symptoms, prior symptom expectations and beliefs are assumed to co-determine symptom perception and are emphasised as triggering, maintaining, and aggravating factors of bodily distress^26,27^. With regard to fatigue as a specific symptom, evidence on placebo effects indicates that verbal suggestions are effective in reducing experienced fatigue and improving physical performance^25^. In terms of nocebo effects, there is first evidence for an effect of negative symptom expectations on self-reported exhaustion^28^, task performance^29^ and the urge to stop a cognitive demanding task^30^ evoked through verbal suggestions in healthy participants. However, the effects of verbal information on perceived fatigue and objective performance or physiological measures were not always consistent so that more evidence is needed to clarify and complement these findings. In particular, investigating whether symptom expectations also affect subjective fatigue levels in a patient group particularly affected by fatigue may contribute to the development of future therapeutic approaches.

As a multidimensional construct, fatigue is often classified into a peripheral and a central dimension^31,32^, even though current self-report questionnaires do not adequately differentiate between both^18^. While peripheral fatigue is characterised by neuromuscular dysfunction in terms of a decline in muscle function and prolonged recovery time (lack of ability), central fatigue refers to a lack of self-motivation characterised by cognitive symptoms such as concentration difficulties and memory impairment (lack of intention)^18,32^. Central fatigue may thus manifest in an urge to stop exerting further effort on a specific task and reduced task engagement^33^. Recent theories of fatigue suggest that subjective fatigue and task disengagement are closely related but conceptually distinct constructs. Fatigue is often conceptualised as an internal signal indicating increasing effort costs or decreasing utility of continued task performance. In contrast, motivation to persist -or the desire to disengage -reflects a decision-making process that integrates multiple inputs, including fatigue, effort valuation, and expected reward^34,35^. Supporting this distinction, Müller et al.^36^ showed that higher momentary fatigue reduces the value of effortful actions, especially when rewards are low, promoting rest decisions. However, individuals may report high fatigue yet remain engaged when sufficiently motivated, or conversely, may disengage even in the absence of strong fatigue signals^37,38^. This dissociation highlights the importance of distinguishing between self-reported mental fatigue and motivational outcomes, even though both contribute to the broader cognitive fatigue profile. In their proposed model for fatigue in PBC, Phaw et al.^39^ distinguish different fatigue endotypes depending on whether cognitive symptoms are present and emphasise that people with fatigue and cognitive symptoms experience the greatest impact on their quality of life. Against this background, this study aims to experimentally investigate how fatigue-inducing (negative instructions) compared to fatigue-reducing instructions (positive instructions) related to a cognitive task affect fatigue and task motivation in patients with PBC. We hypothesised that negative compared to positive task instructions would lead to higher fatigue expectations before the task, higher fatigue levels and a higher urge to stop throughout the task. We did not have any specific hypotheses regarding the effect of task instructions on their task performance.

## Results

### Demographic and baseline characteristics in comparison of both experimental groups

The total sample consisted of *N* = 46 people with PBC with an average age of m = 54.3 years (*SD* = 8.9) and 91% being female. The group with negative instructions did not differ significantly from the group with positive instructions with regard to age, educational level, illness duration, baseline fatigue level, general fatigue expectancy following 10 daily tasks, or fatigue expectancy regarding a 30-minute computer task. There was also no significant group difference for negative or positive affectivity and catastrophising (see Table 1 for an overview of group statistics).

### Manipulation check

The different task instructions did not result in significant group differences in fatigue expectations for the subsequent cognitive task (*t*(44) = -0.64; *p* = .26; Cohen’s *d* = -0.19).

### Correlation analyses

Our correlation analyses across the entire sample revealed that fatigue expectations for the cognitive task after task instructions were positively correlated with participants’ average fatigue (*r*_*s*_ = 0.54, *p* < .001) and motivational urge to stop (*r*_*s*_ = 0.38, *p* = .009) throughout the task. Their average fatigue level during the task, but not their urge to stop, was positively correlated with their baseline fatigue level (*r*_*s*_ = 0.43, *p* = .003), fatigue expectancy following 10 daily tasks (*r*_*s*_ = 0.65, *p* < .001), and catastrophising thoughts (*r*_*s*_ = 0.36, *p* = .015). Positive affectivity was negatively correlated with participants’ average fatigue level (*r*_*s*_ = − 0.46, *p* = .001), but not with their urge to stop. Table 2 outlines the results of all correlation analyses.

### Effects of experimental manipulation on fatigue and motivational urge to stop

**Fig. 1:**
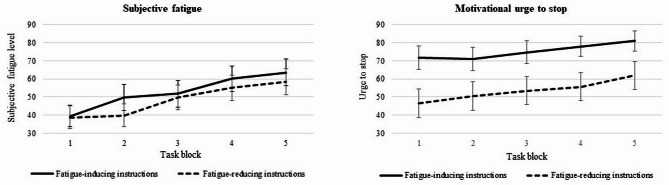
Results of repeated measures ANOVA for fatigue and motivational urge to stop (M ± SE).

After task instructions and prior to the first task block, subjective fatigue levels did not significantly differ between the group with positive (*M* = 32.09; *SD* = 32.95) and negative instructions (*M* = 24.96; *SD* = 25.48) (*t*(44) = -0.82; *p* = .21; Cohen’s *d* = -0.24). With regard to the motivational urge to stop the task, the group with positive instructions reported significantly lower urge to stop at that point (*M* = 42.65; *SD* = 37.08) compared to the group with negative instructions (*M* = 61.65; *SD* = 38.72) (*t*(44) = -1.7; *p* = .048; Cohen’s *d* = 0.24). Across all task blocks, the average mean difference in subjective fatigue levels between the group with positive (*M* = 48.35; *SD* = 30.75) and negative instructions (*M* = 52.94; *SD* = 30.67) was not statistically significant (*t*(44) = -0.51, *p* = .307, Cohen’s *d* = -0.15). For the average motivational urge to stop, the group with positive instructions reported significantly lower urge to stop throughout the task (*M* = 53.58, *SD* = 36.11) compared to the group with negative instructions (*M* = 75.33, *SD* = 21.78) (*t*(44) = -2.31, *p* = .013, Cohen’s *d* = -0.68). Subjective fatigue levels and the motivational urge to stop after each of the five task blocks are outlined in Fig. 1.

Repeated measures ANOVA with subjective fatigue as dependent variable showed a significant main effect of task Block (*F*(3.08,135.64) = 19.12, *p* < .001, partial *η*² = 0.30) implying that fatigue levels significantly increased during the task in both groups. Neither the main effect of Condition (*F*(1,44) = 0.26, *p* = .62, partial *η*² = 0.01) nor the Condition*Block interaction effect (*F*(3.08,135.64) = 0.26, *p* = .53, partial *η*² = 0.02) were significant. The repeated measures ANOVA with urge to stop as dependent variable revealed a significant main effect of Block (*F*(1.61,70.91) = 7.68, *p* < .001, partial *η*² = 0.30), indicating that the motivation to continue with the task decreased throughout the task blocks. There was also a significant main effect of Condition (*F*(1,44) = 5.33, *p* = .03, partial *η*² = 0.12), suggesting that the motivation to continue with the task was lower in the group with negative task instructions. There was no significant Condition*Block interaction effect (*F*(1.61,70.91) = 0.44, *p* = .60, partial *η*² = 0.01).

### Cognitive performance

With regard to task accuracy, there was no significant difference between the group with positive instructions (*M* = 10.57, *SD* = 37.25) and the group with negative instructions (*M* = 5.09, *SD* = 48.14) (*t*(44) = 0.43, *p* = .67, Cohen’s *d* = 0.13). Repeated measures ANOVA with task accuracy as the dependent variable revealed a significant main effect of Block (*F*(2.46,108.41) = 15.89, *p* < .001, partial *η*² = 0.27), indicating an increase in task performance (learning effect) throughout the task. There was neither a significant main effect of Condition (*F*(1,44) = 0.19, *p* = .67, partial *η*² = 0.004) nor a significant Condition*Block interaction effect (*F*(2.46,108.41) = 1.15, *p* = .33, partial *η*² = 0.03).

### Covariate analyses

As positive affectivity and catastrophising showed significant positive correlations with subjective fatigue levels throughout the cognitive task, both variables were examined as potential covariates. The repeated measures ANOVA with subjective fatigue as dependent variable and positive affectivity as covariate did not show a significant main effect of Condition (*F*(1,43) = 0.38, *p* = .53, partial *η*² = 0.01). The main effect of Block was no longer significant (F(3.07,132.11) = 0.67, *p* = .58, partial *η*² = 0.02) and there was no Condition*Block (F(3.07,132.11) = 0.75, *p* = .53, partial *η*² = 0.02) or Block*Positive affectivity (*F*(3.07,132.11) = 0.34, *p* = .81, partial *η*² = 0.01) interaction effect. The repeated measures ANOVA, with catastrophising as a covariate, also did not reveal a significant main effect of Condition (*F*(1,43) = 0.62, *p* = .437, partial *η*² = 0.014), but the main effect of Block was still significant (F(3.03,130.48) = 8.55, *p* < .001, partial *η*² = 0.166). There was no Condition*Block (F(3.03,130.48) = 0.78, *p* = .508, partial *η*² = 0.018) or Block*Catastrophising (F(3.03,130.48) = 0.91, *p* = .439, partial *η*² = 0.021) interaction effect.

Participants’ written answers regarding their awareness of the experimental manipulation did not reveal any indication of suspicion or awareness of the true purpose of the study.

## Discussion

In our experimental study, we examined the effect of expectation-inducing information about a potentially fatiguing vs. activating cognitive task on fatigue expectations, subjective fatigue, the motivational urge to stop doing the task, and task performance in a clinical sample of people with PBC. They received task instructions that either aimed at reducing fatigue expectations (positive instructions) or at inducing fatigue expectations (negative instructions) for the subsequent cognitive task. In both experimental groups, we observed a significant increase in subjective fatigue levels and participants’ motivational urge to stop across the five task blocks. Contrary to our hypothesis and to the results of Lenaert et al. from a healthy sample^30^, both groups did not differ significantly in their fatigue expectations for the subsequent cognitive task after task instructions. Hence, our experimental manipulation may not have been strong enough to create a noticeable difference in participants’ explicit fatigue expectations before the task. Interestingly, prior to the first task block, the group with negative instructions reported significantly higher motivational urge to stop, while both groups did not differ in their subjective fatigue levels. This finding suggests that our task instructions rather affected participants’ motivation to carry out the subsequent cognitive task.

The results of our mixed ANOVA further support this assumption, demonstrating that the group with negative instructions reported higher urge to stop (but not higher fatigue levels) throughout the task relative to the group with positive instructions. Consequently, verbal suggestions may affect patients’ goal-directed behaviour and their motivation to engage in daily tasks. This finding highlights the complexity of the relationship between subjective fatigue and motivational disengagement, suggesting that while these processes are closely related, they may not always change in parallel. Previous research indicates that subjective fatigue can directly influence motivational processes by increasing the perceived cost of effort^34,40^. Moreover, subjective fatigue has been linked to fluctuations in the willingness to exert effort in effort-based decision-making tasks^36,38^. In this line of research, fatigue is conceptualised as a signal, rather than as a direct cause of disengagement. This pattern also aligns with findings in the physical fatigue literature, which show that performance limitations often reflect motivational thresholds rather than purely physiological exhaustion^37^.

In contrast to task motivation, the main effect of experimental condition was not significant for subjective fatigue levels, which is in line with the findings by Lenaert et al.^30^. However, at the descriptive level, the group with positive instructions reported less fatigue compared to the group with negative instructions. Thus, our sample size might have been too small to detect significant group differences in subjective fatigue levels, although it was similar to previous experiments^30,41^. An alternative explanation might be that people tend to keep their fatigue levels in an acceptable range by regulating their exerted effort^42^. In terms of task performance, we observed an improvement of task accuracy over time in both experimental groups indicating learning or adaptation processes during the task, while the main effect of condition was not significant. Findings of Ørskov et al.^43^ suggest that the effect of motivation on task performance may only become apparent after several task blocks which might also apply to our study.

It is also possible that the experimental instructions influenced task-specific self-efficacy, that is, participants’ belief in their ability to complete the task. This may help explain why motivation and the urge to disengage were affected, while subjective fatigue remained unchanged. However, experimental studies suggest that manipulating self-efficacy typically impacts both motivation and perceived exertion^44,45^, making it uncertain that changes in self-efficacy alone would selectively affect disengagement. Future studies should therefore assess self-efficacy directly to disentangle its role and examine how it interacts with fatigue expectations in shaping effort-related outcomes.

At a descriptive level, task performance of the negative instruction group decreased abruptly from the forth to the fifth task block, while task accuracy in the group with positive instructions slightly increased. Previous studies have shown that subjects can maintain their performance despite increasing subjective fatigue levels^46^, which might possibly be compensated by increasing mental effort^47^. Thus, the negative instruction group might not have maintained this compensatory mental effort due to lower motivational levels resulting in a drop in performance at an earlier stage. Future studies should apply longer task periods to further investigate this hypothesis. Repeated measures ANOVA did not identify any significant interaction effects between our between- and within-subject factors or any of the potential covariates.

Correlation analyses showed that fatigue expectations following task instructions correlated positively with participants’ average fatigue levels and motivational urge to stop across all task blocks. This suggests that explicit and task-specific fatigue expectations might affect their motivation for the upcoming task as well as their experienced fatigue. However, this finding can also be interpreted in a way that people adequately anticipated their fatigue level which has an impact on their task-specific motivation. Baseline fatigue and fatigue expectancy following 10 daily tasks also correlated positively with subjective fatigue levels, but not with the urge to stop throughout the task. This reinforces that initial fatigue levels and general fatigue expectancy may affect people’s experienced fatigue during a specific cognitive task. However, it should be noted that participants of our study were not severely affected by fatigue, but rather reported moderate levels, which might be indicative of a selection effect in our sample. Future studies should elaborate further approaches to manipulate fatigue expectations and to examine its effect on subjective fatigue levels.

In clinical practice, health care professionals should communicate with patients carefully with the aim to develop realistic expectations while avoiding unwanted nocebo effects. Regarding the results of our study, the effect of motivation manipulation could be a promising approach to encourage people with PBC to make greater effort and to reduce avoidance behaviour while adjusting existing symptom expectations. The role that symptom expectations and avoidance play in the maintenance of chronic somatic symptoms has recently been emphasised by Nadinda et al.^23^ who proposed an expectancy-avoidance model. Accordingly, people suffering from chronic fatigue, for example in terms of the chronic fatigue syndrome (CFS), might have strong expectations of being fatigued after a specific activity. With the intention of preventing further fatigue, they tend to rest and avoid potentially fatiguing activities, often without first assessing their current fatigue at a somatosensory level. In the long term, consistently avoiding these situations can become dysfunctional, as it prevents people from encountering evidence that challenges their expectations^23^. With regard to people with CFS, there is first evidence that therapeutic interventions aiming to gradually increase duration and intensity of physical exercise are effective in reducing fatigue and disability^48,49^. Similar approaches for people with PBC are currently being developed and tested^50^.

Yet, it is still an open question whether these interventions that mainly address peripheral fatigue might also be transferable to the central fatigue dimension with its primary cognitive symptoms. Findings of previous studies on the effect of task motivation induced by external rewards in cognitive tasks suggest that subjects manage to increase mental effort and maintain task performance when extrinsically motivated. Subjective fatigue levels were inversely correlated with task engagement after reward manipulation^51,52^. Further research is needed to disentangle the interplay between motivational factors and subjective fatigue and its role in the development and persistence of fatigue in people with PBC. A stronger experimental manipulation of fatigue expectations and task motivation could further contribute to a better understanding of this relationship. Such manipulations could incorporate pseudoscientific explanations about the task’s fatigability, personalised feedback about the onset of fatigue, as well as priming procedures (e.g., word or visual priming)^53^.

The results of our study are limited by a relatively small sample size and the overrepresentation of females in our sample, although the sample is representative of the gender ratio in people with PBC. However, previous research suggests that women are more prone to nocebo information that induce somatic symptoms such as pain and nausea while verbally induced placebo responses seem to be more pronounced in male subjects^24,54^. Thus, gender-related differences should be considered carefully when deriving approaches for therapeutic interventions. These gender-specific susceptibility patterns may also have influenced the effectiveness of our verbal fatigue manipulation. Specifically, the higher proportion of women might have increased sensitivity to negative expectations (e.g., in the fatigue-inducing condition) but potentially reduced responsiveness to positively framed instructions (e.g., in the fatigue-reducing condition). Given these differential response tendencies, future studies should aim for more gender-balanced samples or explore gender as a potential moderator in expectation-based paradigms. Moreover, our rather short study protocol limits the implications that can be derived regarding the impact of fatigue expectations and motivation during prolonged task exposure or in everyday life. Future studies should therefore investigate in more detail how the manipulation of motivation and symptom expectations may affect individuals` experienced fatigue in their daily lives.

To conclude, the results of our study indicate that verbal suggestions can influence task engagement in our sample of people with PBC, while there was no effect on their subjective fatigue level. In clinical practice, careful communication that fosters realistic expectations and encourages cognitive activity may help patients to reduce avoidance behaviour and to maintain an appropriate level of activity.

## Methods

### Participants and procedure

Our sample consisted of *N* = 46 people with PBC who participated in the DFG-funded SOMA.LIV research project—a prospective single-center cohort study involving people with both primary sclerosing cholangitis (PSC) and PBC^55^. The sample size was determined based on a power calculation informed by Lenaert et al.^30,41^. Accordingly, a sample size of *N* = 44 was required to detect a small to medium-sized effect between conditions, with 80% power and an *α* error probability of .05. The study received formal ethical approval from the Ethics Committee of the Medical Chamber Hamburg on 21/12/2020 (Processing-No.: 2020-10196-BO-ff). It complies with the World Medical Association Declaration of Helsinki^56^. All participants provided written informed consent to participate in this study. The SOMA.LIV study is part of the interdisciplinary research unit SOMACROSS (RU 5211) entitled ’Persistent SOMAtic Symptoms ACROSS Diseases: From Risk Factors to Modification’^57^. All participants of the present study were recruited at the YAEL-Center for Autoimmune Liver Disease located at the University Medical Center Hamburg-Eppendorf (UKE) in Germany. They had to fulfill the predefined inclusion criteria of the SOMA.LIV study (see Table 3). At 6-months follow-up, participants were contacted and invited to participate in the experimental study part on mental fatigue. People with severe visual and/or hearing impairment were excluded.

### Working memory task

**Fig. 2:**
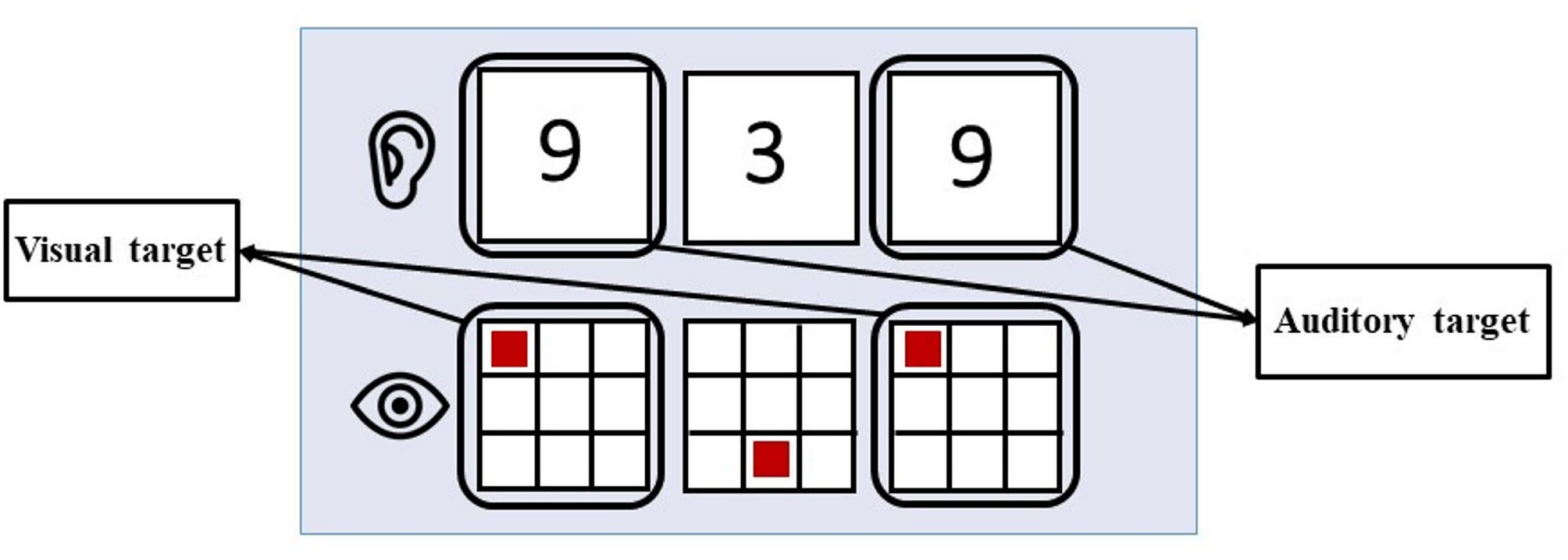
Dual 2-back task with auditory and visual stimuli. Pictograms of ear and eye are retrieved from freeicons.io.

We conducted a dual 2-back task using Presentation^®^ software, version 19.0, Neurobehavioral Systems (California, USA). During the task, visual and auditory stimuli were presented on a computer screen and by headphones (see Fig. 2). Visual stimuli consisted of a red square presented in one of eight possible positions within a three-by-three grid (with a fixation cross at the grid’s center). Participants had to monitor whether a square appeared in the same position as two presentations before (i.e., visual target). Auditory stimuli included numbers from 1 to 9, delivered via headphones. Analogous to the visual targets, participants were required to determine whether the number they heard matched the one presented two numbers back (i.e., auditory target). Each stimulus was displayed for 500 ms with a 2500 ms inter-stimulus interval. Following a brief practice phase, participants completed five task blocks lasting 5 min each with 100 stimulus presentations per block. Each block consisted of eight visual targets, eight auditory targets, four dual targets with both auditory and visual stimuli presented simultaneously, and 80 non-target stimulus presentations. Participants were instructed to react to a visual target, an auditory target, or both by clicking the left mouse button, and to refrain from clicking when no target was presented. This working memory task has previously shown to be effective in inducing mental fatigue within a relatively short time frame^41^. As an indicator of task performance, we focused on the task accuracy measured by the number of correct responses to targets (hits) minus the number of false alarms.

### Measures

#### Fatigue

Before the task, participants rated their current fatigue level, their average fatigue level during the last 24 h and their worst fatigue level during the last 24 h on a Visual Analogue Scale (VAS) from 0 (no fatigue) to 10 (extreme fatigue). The three items were averaged to indicate people’s baseline fatigue level. Before the first task block and after each of the five task blocks, participants again rated their current fatigue level on a VAS presented on the computer screen (“How tired do you feel right now?”) from 0 (not at all) to 100 (extremely).

#### Fatigue expectancy

 Before performing the working memory task, participants rated their expected fatigue levels following 10 daily tasks (a 10-minute walk on a flat surface, 15-minute ironing, a 2-minute run, 30-minute reading, 2 floors of stair climbing and descending, washing dishes, preparing a meal, driving a car for 30 min, 30-minute computer work and 15-minute gardening)^58^ on a VAS from 0 (no fatigue) to 10 (severe fatigue) to assess their overall fatigue expectancy. The item on 30-minute computer work further served as an indicator of participants’ implicit fatigue expectations for the subsequent 30-minute computer task.

#### Motivation

A second VAS was presented before the first and after each of the five task blocks to assess people’s motivational urge to stop doing the task (“To what extent do you want to stop doing this task?”) from 0 (not at all) to 100 (extremely).

#### Manipulation check

After receiving detailed task instructions that included experimental manipulation (negative vs. positive task instructions), participants were asked to rate their fatigue expectation for the upcoming working memory task on the computer screen.

#### Negative and positive affectivity

We measured negative and positive affect using the German Version of the Positive and Negative Affectivity Schedule (PANAS trait-version)^59,60^. This questionnaire consists of 20 items on two scales assessing a person’s general positive (e.g., ‘enthusiastic’) and negative (e.g., ‘distressed’) traits on a 5-point Likert scale. The sum score of the negative trait items represents the total negative affect with higher values indicating higher negative affectivity. The score of the total positive affect is formed analogously.

#### Catastrophising

 Two items of the Coping Strategies Questionnaire - Catastrophising Subscale (CSQ-CAT)^52^ assessed people’s catastrophising thoughts towards physical symptoms.

#### Manipulation awareness 

After finishing the cognitive task, participants answered two questions in written form on their assumption regarding the aim of this experiment and whether they assumed a link between the task instructions at the beginning and their well-being or performance during the task.

### Procedure

The experimental paradigm used in our study was based on Lenaert et al.^30^ who investigated whether nocebo information compared to neutral task instructions about a cognitive task contributed to the experience of fatigue and the motivational urge to stop in healthy participants. After giving their informed consent, participants of our study were randomly assigned to one of two experimental conditions (negative vs. positive task instructions) and filled in the questionnaire to assess their baseline fatigue level. Next, participants received written task instructions about the subsequent working memory task. They were informed that our study investigates symptom perception during a working memory task of 5 task blocks on the computer. Participants assigned to the fatigue-inducing condition were informed that *“…participants in previous studies had evaluated the task as cognitively demanding and had experienced fatigue following the task. It is therefore possible that your performance will deteriorate over the course of the task and that you will feel increasingly exhausted”* (negative instructions). Participants assigned to the fatigue-reducing condition were told that *“…participants in previous studies had experienced an improvement in their ability to concentrate and pay attention during the course of the task. It is therefore possible that your performance will improve over the course of the task and that you will feel increasingly activated”* (positive instructions). All task instructions and information were repeated verbally. Before participants started with a brief practice cycle, they were asked about their fatigue expectation for the upcoming task (manipulation check). Remaining questions were clarified before participants started with the five task blocks. After finishing the task, they were fully debriefed about the study’s objective and received financial compensation for their participation via bank transfer. Our study was preregistered at the Open Science Framework (https://osf.io/xz23f/).

### Statistical analysis

Statistical analyses were conducted using SPSS version 25, with an α error probability set at 0.05. Differences in clinical, demographic and baseline variables (age, education, illness duration, baseline fatigue level, fatigue expectancy, negative and positive affectivity, catastrophising) between conditions were assessed using independent sample t-tests and chi-squared test. Differences in fatigue expectations following experimental manipulation as well as current fatigue levels and the motivational urge to stop before the first task block were also assessed via independent sample t-tests. Bivariate correlation analyses were performed to explore associations between baseline characteristics, potential covariates and primary outcomes (subjective fatigue and motivational urge to stop). Repeated measures analysis of variance (ANOVA) were run to examine the effect of the experimental condition on fatigue levels, motivational urge to stop and task performance across the five task blocks. Task blocks (1–5) thereby served as the within-subjects factor, and condition (positive vs. negative instructions) as the between-subjects factor. In case of significant correlations between potential covariates and primary outcomes (subjective fatigue and motivational urge to stop), ANOVAs were rerun with the respective covariates. Greenhouse-Geisser corrections were applied when Mauchly’s test of sphericity indicated a violation of the sphericity assumption.

**Table 1:** Clinical, demographic and baseline characteristics.

	Fatigue-inducing task instruction	Fatigue-reducing task instruction			
	*n* = 23 (22 female)	*n* = 23 (20 female)			
Variable	(*M* ± *SD*)/*n* (%)	(*M* ± *SD*)/*n* (%)	*t/ꭕ2*	*df*	*p* -value
Age	52.83 (7.77)	55.7 (9.91)	1.10	44	0.277
Education					
School without graduation	1 (4.3%)	0 (0.0%)	2.20	3	0.532
≤ 10 years	9 (39.1%)	11 (47.8%)			
> 10 years	12 (52.2%)	12 (52.2%)			
Other	1 (4.3%)	0 (0.0%)			
Illness duration PBC (years)	9.3 (6.85)	7.52 (4.56)	-1.04	44	0.305
Baseline Fatigue	5.28 (2.03)	4.88 (2.51)	− 0.58	44	0.564
Fatigue expectancy following 10 daily tasks	2.53 (1.82)	2.61 (1.89)	0.16	44	0.875
Fatigue expectancy following 30-minute computer task	3.26 (2.6)	3.48 (2.73)	0.28	44	0.783
Fatigue expectation after task instructions	43.91 (29.8)	38.35 (29.56)	− 0.64	44	0.264^a^
Positive affectivity (PANAS)	3.26 (0.6)	3.24 (0.75)	− 0.07	44	0.948
Negative affectivity (PANAS)	1.65 (0.44)	1.68 (0.37)	0.29	44	0.772
Catastrophising (CSQ-CAT)	2.39 (2.91)	2.87 (2.38)	0.61	44	0.546

PANAS = Positive and Negative Affectivity Schedule; CSQ-CAT = Coping Strategies Questionnaire - Catastrophising Subscale (only 2 items)

^a^ One-tailed t-test

**Table 2:** Pearson and spearman correlation coefficients of questionnaires and outcome variables.

Measure	1	2	3	4	5	6	7	8	9	10
1. Average fatigue level^a^		0.44**	− 0.07	0.43**	0.65**	0.56**	0.54**	− 0.46**	0.21	0.36*
2. Average urge to stop^a^	0.48**		0.06	0.16	0.23	0.23	0.38**	− 0.01	− 0.04	0.05
3. Total task accuracy^a^	− 0.14	− 0.00		− 0.01	− 0.04	− 0.01	0.03	0.01	0.08	− 0.19
4. Baseline fatigue	0.47**	0.24	− 0.05		0.59**	0.44**	0.26	− 0.36*	0.19	0.42**
5. Fatigue expectancy (10 items)^a^	0.67**	0.26	− 0.08	0.59**		0.84**	0.40**	− 0.42**	0.33*	0.45*
6. Fatigue expectancy (30-minute computer work)^a^	0.60**	0.25	− 0.01	0.47**	0.84**		0.45**	− 0.38*	0.32*	0.31*
7. Fatigue expectation for cognitive task (after task instruction)	0.51**	0.35*	− 0.01	0.28	0.40**	0.46**		− 0.36*	0.20	0.15
8. Positive affectivity	− 0.50**	− 0.07	0.03	− 0.39**	-51**	− 0.44**	− 0.40**		− 0.24	− 0.16
9. Negative affectivity	0.25	0.00	0.01	0.20	0.30*	0.31*	0.20	− 0.29*		0.35*
10. Catastrophising^a^	0.371*	0.09	− 0.47**	0.38**	0.44**	0.27	0.11	− 0.14	0.29	

The bottom left half of the table contains Pearson´s correlation coefficients, the upper right half shows Spearmans`s correlation coefficients.

^a^ Variable not normally distributed as indicated by a Shapiro-Wilk test of *p* < .05

* *p* < .05 (two-tailed)

** *p* < .01 (two-tailed)

**Table 3:** Participant selection criteria.

Inclusion criteria	Exclusion criteria
Clinical diagnosis of PBC according to generally accepted criteria [5]	⋅ Advanced cirrhosis (defined by Child Pugh A score ≥ 8) or decompensated liver disease
Age ≥ 18 years	⋅ History or presence of other concomitant liver disease (especially autoimmune hepatitis or chronic viral hepatitis B or C)
Sufficient oral and written German language proficiency	⋅ Presence of clinically significant untreated intercurrent medical condition associated with fatigue (i.e. hypothyroidism, anaemia, fibromyalgia, rheumatoid arthritis, systemic lupus erythematosus, active IBD and manifest depression)
Informed consent	⋅ Serious illness requiring immediate intervention
	⋅ Florid psychosis
	⋅ Substance abuse disorder
	⋅ Acute suicidality

PBC = Primary biliary cholangitis; IBD = Inflammatory Bowel Disease

## Data Availability

The data that support the findings of this study are available on request from the corresponding author, LB. The data are not publicly available to ensure the privacy of the research participants.
